# Editorial: Registered reports on the role of representational competencies in multimedia learning and learning with multiple representations

**DOI:** 10.3389/fpsyg.2023.1260833

**Published:** 2023-08-11

**Authors:** Sarah Malone, Ana Susac, Jochen Kuhn, Stefan Küchemann

**Affiliations:** ^1^Department of Education, Saarland University, Saarbrücken, Germany; ^2^Department of Applied Physics, Faculty of Electrical Engineering and Computing, University of Zagreb, Zagreb, Croatia; ^3^Faculty of Physics/Chair of Physics Education, Ludwig-Maximilians-Universität München (LMU Munich), Munich, Free State of Bavaria, Germany

**Keywords:** multiple representations, science technology engineering mathematics (STEM), registered report, representational competence, multimedia learning

External representations are information carriers that commonly manifest in various formats, such as text, graphs, or formulas, displayed, for example, in educational resources, such as textbooks or computer-based learning programs. The use of more than one external representation, i.e., the combination of various types of representations for conveying information related to a specific topic or concept, is called multimedia learning (mostly for text-picture combinations; Mayer and Fiorella, 2022) or learning with multiple external representations (Ainsworth, 2006). There is evidence suggesting that learning with multiple representations, rather than single representations, can foster learning (e.g., Lee et al., 2006; Eitel et al., 2013) and problem-solving (e.g., Lindner et al., 2017; Ott et al., 2018).

The term *representational competencies* or *competence* refers to the ability to select, analyze, and utilize external representations to support claims, explain concepts, make predictions, and draw inferences. Representational competencies involve specific skills, such as transforming one representation into another, selecting appropriate representations, and understanding how different representations convey information in distinct ways (Kozma and Russell, 1997). Representational competencies have been found to be positively correlated with conceptual knowledge (Edelsbrunner et al., 2023).

There are different assumptions about how representational competencies might affect learning processes related to multiple external representations. Learning with multiple representations can place high demands on learners, since they are required to handle complex, domain-specific representations (e.g., Stieff et al., 2011). It is assumed that a representation dilemma can occur when learners are offered representations that include essential information but fail to benefit from them due to their lack of representational competencies (Rau, 2017). Accordingly, one could perceive representational competence as a prerequisite for successful learning with multiple representations. However, there is also the assumption that representational competence and content or conceptual knowledge can be acquired simultaneously through appropriate instruction. Moreover, first evidence suggests that there may be gender differences in the strength of the relationship between representational competencies and conceptual knowledge, suggesting gender-specific interventions (Edelsbrunner et al., 2023).

Despite some initial intriguing findings coming exclusively from Science, Technology, Engineering, and Math (STEM) subjects, research on the role of representational competencies for learning with external representations is still in its infancy. This Research Topic aims to bring together research that examines the interplay of representational competencies, associated skills and aptitudes, and learning with multiple representations.

All articles are Registered Reports. The planned and pre-registered studies are published in this Volume 1 of the Research Topic as stage 1 papers. The corresponding stage 2 papers will be published in Volume 2 of the Research Topic upon their completion.

Volume 1 includes four articles from various STEM disciplines.

The stage 1 paper “*Unraveling the relation between representational competence and conceptual knowledge across four samples from two different countries*” by Edelsbrunner and Hofer intends to study the statistical relation of representational competence and conceptual knowledge in physics education. The authors aim to further investigate this relationship by re-analyzing data from a previous study that found a significant positive correlation between these two constructs in the context of vector-field representations and electromagnetism (Küchemann et al., 2021). The data, collected from 515 undergraduate students across two countries, will be used to examine whether the relationship between representational competence and conceptual knowledge varies across different sub-samples. The goal is to understand the generalizability of this relationship and to derive hypotheses for potential moderating factors that can be further examined in future research.

Another paper that deals with physics education is the stage 1 paper by Hahn and Klein, titled “*The impact of multiple representations on students' understanding of vector field concepts: implementation of simulations and sketching activities into lecture-based recitations in undergraduate physics*.” The research aims to investigate the effectiveness of multi-representational learning tasks in enhancing students' understanding of vector calculus concepts in the context of electrodynamics. The developed tasks focus on the visual interpretation of vector field diagrams and incorporate different representational activities such as sketching and using interactive simulations. In their field study, the authors will compare the impact of these multi-representational tasks to traditional calculation-based tasks on university students' mathematical as well as conceptual understanding of vector calculus concepts and on their cognitive load.

In their stage 1 paper titled “*Analyzing and supporting mental representations and strategies in solving Bayesian problems*,” Sirock et al. focus on the challenges associated with solving Bayesian problems by means of different types of external representations. The authors provide a comprehensive theoretical framework for the assumed underlying cognitive processes and analyze various approaches to support these processes. The goal of their study is to examine how different types of representations, namely a unit square and a 2 x 2 table, can impact the solution of Bayesian problems and students' active and passive cognitive load compared with Bayesian formula. Furthermore, the study will examine to what extent different aptitude variables (prior knowledge, spatial abilities, and logical abilities) moderate the influence of the representational format. The authors will investigate their research questions using a within-subjects design.

Ripsam and Nerdel present a stage 1 paper titled “*Augmented reality for chemistry education to promote the use of chemical terminology in teacher trainings*.” The purpose of their planned empirical study is to investigate the potential impact of augmented reality instruction on the development of representational competencies, i.e., chemistry teachers' reflective use of chemical terminology. The authors hypothesize that the presentation of multiple representations through augmented reality during experimentation can eliminate the issue of split attention and thereby enhance learning. To explore this hypothesis, the study will compare Group 1 using AR on a tablet, Group 2 using AR on a Hololens, and a control group using a simulation-based learning environment on a tablet. The study will capture the chemistry terminology of participating teachers using pre- and post-surveys. Additionally, the researchers will employ thinking-aloud protocols and evaluate the acquired use of chemical terminology using a qualitative data analysis software.

## Author contributions

SM: Conceptualization, Writing—original draft. AS: Conceptualization, Writing—original draft. JK: Conceptualization, Writing—original draft. SK: Conceptualization, Writing—original draft.
